# Five-year follow-up of patients with relapsed/refractory mantle cell lymphoma treated with anti-CD19 CAR T-cell therapy in ZUMA-2, Cohorts 1 and 2

**DOI:** 10.1186/s13045-026-01797-4

**Published:** 2026-04-27

**Authors:** Javier Muñoz, Frederick L. Locke, Patrick M. Reagan, Andre Goy, Caron A. Jacobson, Brian T. Hill, John M. Timmerman, Ian W. Flinn, David B. Miklos, John M. Pagel, Marie José Kersten, Edouard Forcade, Max S. Topp, Roch Houot, Amer Beitinjaneh, Dan Zheng, Mengru Chang, Wangshu Zhang, Rhine R. Shen, Rita Damico Khalid, Ioana Kloos, Michael L. Wang

**Affiliations:** 1 Banner MD Anderson Cancer Center, Gilbert, AZ USA; 2 Mayo Clinic, Pheonix, AZ USA; 3 Moffitt Cancer Center, Tampa, FL USA; 4 University of Rochester School of Medicine, Rochester, NY USA; 5 John Theurer Cancer Center, Hackensack, NJ USA; 6 Dana-Farber Cancer Institute, Boston, MA USA; 7 Cleveland Clinic Foundation, Cleveland, OH USA; 8 UCLA David Geffen School of Medicine, Los Angeles, CA USA; 9 Tennessee Oncology & One Oncology, Nashville, TN USA; 10 Stanford University, Stanford, CA USA; 11 Swedish Cancer Institute, Seattle, WA USA; 12 Amsterdam UMC, University of Amsterdam Cancer Center, Amsterdam, Netherlands; 13 Service d’Hématologie Clinique et Thérapie Cellulaire, CHU Bordeaux, Pessac, France; 14 Medizinische Klinik und Poliklinik II, Universitätsklinikum Würzburg, Würzburg, Germany; 15 CHU Rennes, Université Rennes, Inserm & EFS, Rennes, France; 16 University of Miami, Miami, FL USA; 17 Kite, a Gilead Company, Santa Monica, CA USA; 18 The University of Texas MD Anderson Cancer Center, Houston, TX USA

**Keywords:** Brexu-cel, CAR T-cell therapy, Mantle cell lymphoma, ZUMA-2, Long-term follow-up

## Abstract

**Background:**

Treatment with brexucabtagene autoleucel (brexu-cel), an autologous anti-CD19 chimeric antigen receptor (CAR) T-cell therapy, demonstrated a high objective response rate (93%) and complete response rate (67%) in 60 patients with relapsed/refractory mantle cell lymphoma (R/R MCL) treated in the pivotal ZUMA-2 Cohort 1 study. Subsequently, brexu-cel was approved in the United States and European Union for the treatment of adults with R/R MCL (after ≥ 2 prior therapies in the European Union). Here we report 5-year outcomes from the pivotal ZUMA-2 Cohort 1 study (*N* = 68), as well as two previously unpublished ZUMA-2 data sets, long-term outcomes in 10 patients who received axi-cel in Cohort 1 and in 14 patients who received a lower dose of brexu-cel in Cohort 2.

**Methods:**

The primary endpoint for all cohorts of ZUMA-2 was objective response rate. Key secondary endpoints included duration of response (DOR), overall survival (OS), and safety. Patients could transition to a long-term follow-up study after 24 months for monitoring of survival and select adverse events possibly related to brexu-cel. Patients in Cohort 1 received a single infusion of 2 × 10^6^ anti-CD19 CAR T cells/kg (axi-cel or brexu-cel). Patients in Cohort 2 received 0.5 × 10^6^ anti-CD19 CAR T cells/kg (brexu-cel).

**Results:**

Median follow-up for the pivotal cohort (*N* = 68) was 67.8 months (range, 58.2–88.6) with a median DOR of 36.5 months (*n* = 60), per investigator review. Median OS was 46.5 months (95% CI, 24.5–60.2; *N* = 68) and was 60.2 months (95% CI, 42.8-not estimable) in patients with complete response (*n* = 46). The 5-year incidence of cumulative relapse-related and non-relapse–related mortality was 40% (24/60) and 22% (13/60) in responders, respectively. Descriptive outcomes for axi-cel–treated patients (*N* = 10) and Cohort 2 (*N* = 14) are reported herein. No Grade 5 cytokine-release syndrome or neurologic events, subsequent T-cell malignancies, or new safety signals were reported in any patient.

**Conclusions:**

Patients in ZUMA-2 continued to have durable responses after 5 years of follow-up with predictable long-term safety, supporting the continued use of brexu-cel in R/R MCL. Interpretations of outcomes in axi-cel–treated patients and Cohort 2 are not feasible due to small patient numbers and unmatched baseline characteristics.

**Trial registration:**

NCT02601313 and NCT05041309.

**Supplementary Information:**

The online version contains supplementary material available at 10.1186/s13045-026-01797-4.

## Introduction

Brexucabtagene autoleucel (brexu-cel) is an autologous anti-CD19 chimeric antigen receptor (CAR) T-cell therapy that has been approved in the United States for the treatment of adults with relapsed/refractory mantle cell lymphoma (R/R MCL) since July of 2020 and in the European Union (EU) for adults with R/R MCL after receiving ≥ 2 prior systemic treatments including a Bruton tyrosine kinase inhibitor (BTKi) since December of 2020. [1] Approval was based on the high objective response rate (ORR; 93%) and complete response (CR) rate (67%) observed for the first 60 patients with R/R MCL treated in the pivotal Cohort 1 ZUMA-2 study (NCT02601313). [2] Median time from infusion to CR was 3.0 months (range, 0.9–9.3). After 3 years of median follow-up, brexu-cel demonstrated an ORR of 91%, a CR rate of 68%, and a median duration of response (DOR) and overall survival (OS) of 28.2 and 46.6 months, respectively, in all treated patients in ZUMA-2 Cohort 1 [3].

Beyond the pivotal dose of brexu-cel, two other patient groups received different treatments in ZUMA-2. The first 10 patients in Cohort 1 of ZUMA-2 received axicabtagene ciloleucel (axi-cel) and 14 patients received a lower dose (0.5 × 10^6^ anti-CD19 CAR T cells/kg) of brexu-cel in Cohort 2 of ZUMA-2. Axicabtagene ciloleucel (axi-cel), manufactured using unfractionated peripheral blood mononuclear cells (PBMC) as a starting material, is an anti-CD19 CAR T-cell therapy for patients with lymphomas associated with limited circulating tumor cells, and had previously demonstrated a high response rate and durable responses in patients with large B-cell lymphoma in ZUMA-1 (NCT02348216). [4] The first 10 patients in ZUMA-2 (R/R MCL) received axi-cel, but due to high levels of CD-19 circulating tumor cells detected in the peripheral blood of these patients, all subsequent patients in ZUMA-2 received brexu-cel, which is an anti-CD19 CAR T-cell therapy manufactured using T cells isolated from PBMC, intended for patients with leukemias or lymphomas with potentially high levels of CD19-expressing circulating tumor cells.

Additionally, after 28 patients received brexu-cel at a dose of 2 × 10^6^ anti-CD19 CAR T cells/kg (same dose as axi-cel) in Cohort 1, an interim analysis found that CAR T-cell expansion and rates of Grade ≥ 3 neurologic events were higher in these patients than what was observed in ZUMA-1; thus, Cohort 2 was established to determine if a lower dose of brexu-cel (0.5 × 10^6^ anti-CD19 CAR T cells/kg) would reduce the risk of severe neurologic events. However, evaluation of the first 14 patients in Cohort 2 found that CAR T-cell expansion was less robust than in Cohort 1, whereas severe adverse events (AEs) occurred at similar rates. Concurrently, outcomes for the first 28 patients in Cohort 1 demonstrated a high ORR and 6-month DOR rate in a heavily pretreated population with manageable safety. Together, these findings led to the decision to select the Cohort 1 dose as the optimal and pivotal dose for ZUMA-2; thus, Cohort 2 enrollment was concluded with only 14 patients treated at the lower dose. Outcomes in axi-cel–treated patients and those who received the lower dose of brexu-cel have not been reported publicly to date, given the small patient numbers.

All patients in ZUMA-2 who received an infusion of brexu-cel or axi-cel had the opportunity to transition to a long-term follow-up (LTFU) study (NCT05041309), where they were monitored for late-onset–targeted or serious AEs (SAEs) possibly related to anti-CD19 CAR T-cell treatment. Limited data are available for long-term efficacy and safety of patients treated with CAR T-cell therapies; however, this type of therapy has the potential to produce durable responses and long-term survival. [5] Consequently, it is important to continue to assess and characterize long-term outcomes of patients who receive CAR T-cell therapy to understand the full benefits and risks associated with these types of therapies. Therefore, herein, we report updated 5-year efficacy and safety outcomes of brexu-cel–treated patients in ZUMA-2 Cohort 1. Furthermore, for the first time, we report primary efficacy and pharmacokinetic outcomes as well as descriptive long-term efficacy and safety outcomes in Cohort 2, and descriptive long-term safety outcomes for patients in Cohort 1 who were treated with axi-cel.

## Methods

### Patients and trial design 

ZUMA-2, is a multicohort, multicenter, Phase 2, open-label study conducted across 26 sites in North America and Europe that evaluated the safety and efficacy of anti-CD19 CAR T-cell therapy in patients with R/R MCL. [2] Detailed study methods were previously reported. [2] Briefly, eligible patients were ≥ 18 years of age and had received up to 5 prior regimens for MCL, including an anthracycline or bendamustine-containing chemotherapy, an anti-CD20 monoclonal antibody, and BTKi therapy. After completion of ZUMA-2, patients could transition to the LTFU study, where they were and will continue to be monitored for late-onset AEs possibly related to brexu-cel for up to 15 years.

Trial protocols and statistical analysis plans were developed by Kite, a Gilead Company, in collaboration with the investigators (Supplemental Appendix). At each study site, the institutional review board or independent ethics committee approved the study protocol, and all patients provided written informed consent. The studies were conducted in accordance with principles of the Declaration of Helsinki. ZUMA-2 and the LTFU study are registered at ClinicalTrials.gov (NCT02601313 and NCT05041309, respectively).

**Treatment**.

Patients underwent leukapheresis and optional bridging therapy (dexamethasone, ibrutinib, or acalabrutinib) per physician’s discretion. Bridging therapy was recommended for patients with high disease burden at screening (> 25% marrow involvement and/or ≥ 1000 leukemic phase mantle cells/mm^3^ in peripheral blood) and was administered after leukapheresis and at least 5 days before the start of lymphodepleting chemotherapy. Lymphodepleting chemotherapy was given on Days −5, −4, and −3 (fludarabine 30 mg/m^2^/day and cyclophosphamide 500 mg/m^2^/day). A single infusion of anti-CD19 CAR T cells was administered on Day 0. In Cohort 1, patients were administered 2 × 10^6^ anti-CD19 CAR T cells/kg of axi-cel (first 10 patients dosed) or brexu-cel (*n* = 68 dosed). In Cohort 2, patients were administered brexu-cel at a dose of 0.5 × 10^6^ anti-CD19 CAR T cells/kg. Hospitalization was required for at least 7 days after anti-CD19 CAR T-cell infusion.

### Endpoints and assessments

The ZUMA-2 primary endpoint was ORR, defined as CR rate plus partial response (PR) rate, as assessed by independent radiology review committee (IRRC) per the Lugano classification.[6] Secondary endpoints included DOR, defined as the time from first objective response to disease progression or death; best objective response (BOR) to treatment, defined as the incidence of CR, PR, stable disease (SD), progressive disease (PD), or unevaluable; and investigator-assessed ORR. For Cohort 1, investigator-assessed ORR was defined as the incidence of CR or PR per revised International Working Group Response Criteria for Malignant Lymphoma [7]; and for Cohort 2, it was defined as CR or PR per the Lugano classification. [6] Other secondary endpoints were progression-free survival (PFS); OS; AEs (after 3 months, only targeted AEs, ie, neurologic events, hematologic events, infections, graft-versus-host disease, autoimmune disorders, and subsequent malignancies, were monitored; Supplemental Appendix) and clinically significant changes in laboratory values; incidence of anti-CD19 CAR antibodies, levels of anti-CD19 CAR T-cell levels in blood; and serum cytokine levels. CAR T-cell levels in blood and product characteristics were analyzed using previously described methods. [2] B-cell levels in PBMCs were analyzed using previously described methods. [8].

The primary endpoint of the LTFU study was to assess the occurrence of late-onset–targeted AEs/SAEs possibly related to gene-modified cells (Table S1). Key LTFU secondary endpoints were OS, causes of death, and rates of replication-competent retrovirus/replication-competent lentivirus.

### Statistical analysis

Brexu-cel administered at a dose of 2 × 10^6^ anti-CD19 CAR T cells/kg was determined to be the optimal dose and, thus, Cohort 1 was completed as the registrational ZUMA-2 study. The primary analysis of Cohort 1 was performed after 60 patients were treated with brexu-cel and were evaluated for response, as previously reported. [2] Additional ZUMA-2 reports with longer follow-up included all 68 dosed patients. [3] patients. Analyses herein of patients in Cohort 1 who were treated with axi-cel and patients in Cohort 2 are exploratory and descriptive in nature, with no hypotheses tested, as these cohorts did not reach full enrollment.

Time-to-event endpoints were analyzed with Kaplan-Meier estimates and 2-sided 95% confidence intervals. DOR and ongoing responder analyses were assessed per investigator review given all patients were beyond the prespecified IRRC response assessment period at the time of this LTFU analysis. Safety analyses included all patients treated with axi-cel and brexu-cel whereas efficacy analyses included all patients treated with any dose of brexu-cel in ZUMA-2 Cohorts 1 and 2.

## Results

### Patients

In Cohort 1, 14 patients were enrolled to receive axi-cel from November 24, 2015, to October 19, 2016, with 10 patients treated; 74 patients were enrolled to receive brexu-cel from October 24, 2016, to April 16, 2019, with 68 patients treated (Figure S1 ). As of October 5, 2023, the median follow-up in axi-cel–treated patients was 85.5 months (range, 79.4–93.7) with 5 patients (50%) alive at data cutoff (Figure S2). As of April 1, 2024, the median follow-up in brexu-cel–treated patients was 67.8 months (range, 58.2–88.6) with 24 patients (35%) alive (2 withdrew consent and 1 was lost to follow-up) and 44 patients (65%) who had died by data cutoff (Figure S3).

In Cohort 2, 17 patients were enrolled from January 2, 2018, to May 15, 2018; 14 patients were treated, with a median follow-up of 72.3 months (range 70.1–74.3) at time of the 5-year analysis data cutoff date, April 1, 2024. Of the 14 patients treated in Cohort 2, 8 patients (57%) were still alive (2 withdrew consent and 1 was lost to follow-up) and 6 patients (43%) had died as of the 5-year data cutoff date (Figure S4).

As of April 1, 2024, a total of 27 patients who were treated with brexu-cel (Cohort 1, *n* = 23; Cohort 2, *n* = 4) rolled over to the LTFU study with an actual median follow-up of 65.8 months (range, 46.9–88.6). Two patients in the LTFU study had died by the time of the analysis, both from Cohort 1 (Figure S3).

Table 1 reports demographics and baseline characteristics of patients treated with axi-cel and brexu-cel in Cohorts 1 (as previously reported) and 2, as well as characteristics by ongoing response status in Cohort 1 at time of the 5-year analysis. [2] Generally, baseline characteristics were similar across cohorts though there were notable differences in median age, morphology, and proportion who received bridging therapy. In Cohort 1, median age was 67.0 years (range, 43–75) for patients treated with axi-cel and 65.0 years (range, 38–79) for patients treated with brexu-cel. The median age for patients in Cohort 2 was 61.5 years (range, 52–73). High-risk features were common as ≥ 50% of patients had a high or intermediate simplified Mantle Cell Lymphoma International Prognostic Index (s-MIPI) score across all groups. For patients treated with brexu-cel in Cohorts 1 and 2, 25% and 43% of patients had blastoid morphology, and 63% and 71% of patients had Ki-67 expression ≥ 30%, respectively. Median number of prior therapies was 3 for patients treated with brexu-cel in Cohorts 1 and 2. Forty-three percent of patients treated with brexu-cel in Cohorts 1 and 2 and 40% of patients treated with axi-cel had prior autologous stem cell transplantation (ASCT). Among patients treated with brexu-cel, 37% in Cohort 1 and 50% in Cohort 2 received bridging therapy. Patient characteristics were largely similar across ongoing responder subgroups in Cohort 1, although a smaller proportion of patients with ongoing response had prior bridging therapy, prior bendamustine therapy, or prior lenalidomide therapy.

**Table 1:** Demographics and baseline disease characteristics for all patient groups (safety analysis set)

Characteristic	Cohort 1					Cohort 2^d^ (*N* = 14)
	Axi-cel-treated^a^ (*N* = 10)	Brexu-cel–treated (*N* = 68)				
		Total^b^ (*N* = 68)	Ongoing responders^c^ (*n* = 17)	Relapsed responders^c^ (*n* = 36)	Non- responders^c^ (*n* = 8)	
Male, n (%)	9 (90)	57 (84)	15 (88)	31 (86)	6 (75)	11 (79)
Median age, y (range) ≥ 65 y	67.0 (43–75) 6 (60)	65.0 (38–79) 39 (57)	65.0 (55–79) 9 (53)	65.0 (38–75) 22 (61)	65.0 (54–73) 5 (63)	61.5 (52–73) 3 (21)
ECOG performance status, n (%) 0 1	8 (80) 2 (20)	44 (65) 24 (35)	12 (71) 5 (29)	23 (64) 13 (36)	4 (50) 4 (50)	7 (50) 7 (50)
Morphological characteristics, n (%) Classical MCL – diffuse Classical MCL – nodular Classical MCL – other Pleomorphic MCL Blastoid MCL Other^e^ Unknown	2 (20) 2 (20) 3 (30) 0 2 (20) 0 1 (10)	20 (29) 10 (15) 6 (9) 4 (6) 17 (25) 1 (1) 10 (15)	2 (12) 5 (29) 1 (6) 2 (12) 2 (12) 0 5 (29)	11 (31) 3 (8) 5 (14) 2 (6) 11 (31) 1 (3) 3 (8)	5 (63) 0 0 0 3 (38) 0 0	4 (29) 1 (7) 0 2 (14) 6 (43) 1 (7) 0
Ki-67 (%) IHC by central laboratory^f^, n (%) ≥ 30% ≥ 50%	– –	43 (63) 37 (54)	11 (65) 10 (59)	21 (58) 18 (50)	6 (75) 4 (50)	10 (71) 9 (64)
T(11;14) FISH by central laboratory, n (%) Detected Not detected Missing	6 (60) 1 (10) 3 (30)	48 (71) 2 (3) 18 (26)	12 (71) 1 (6) 4 (24)	25 (69) 1 (3) 10 (28)	6 (75) 0 2 (25)	10 (71) 2 (14) 2 (14)
Disease stage, n (%) I II III IV	0 2 (20) 1 (10) 7 (70)	0 2 (3) 8 (12) 58 (85)	0 0 3 (18) 14 (82)	0 1 (3) 3 (8) 32 (89)	0 0 1 (13) 7 (88)	0 0 1 (7) 13 (93)
Extranodal disease present, n (%)	8 (80)	38 (56)	11 (65)	19 (53)	4 (50)	10 (71)
LDH relative to upper limit, n (%) LDH < 0.67 ULN 0.67 ULN ≤ LDH < ULN ULN ≤ LDH < 1.5 ULN 1.5 ULN ≤ LDH Missing	1 (10) 4 (40) 2 (20) 2 (20) 0	16 (24) 24 (35) 15 (22) 11 (16) 2 (3)	3 (18) 6 (35) 5 (29) 2 (12) 1 (6)	8 (22) 13 (36) 6 (17) 8 (22) 1 (3)	2 (25) 4 (50) 1 (13) 1 (13) 0	5 (36) 3 (21) 2 (14) 3 (21) 1 (7)
Simplified MIPI, n (%) Low risk Intermediate risk High risk Missing	3 (30) 5 (50) 1 (10) 0	28 (41) 29 (43) 9 (13) 2 (3)	7 (41) 7 (41) 2 (12) 1 (6)	13 (36) 16 (44) 6 (17) 1 (3)	2 (25) 5 (63) 1 (13) 0	6 (43) 4 (29) 3 (21) 1 (7)
Relapsed/refractory subgroup, n (%) Relapse after autologous stem cell transplantation Refractory to last MCL therapy Relapse after last MCL therapy	4 (40) 5 (50) 1 (10)	29 (43) 27 (40) 12 (18)	8 (47) 5 (29) 4 (24)	15 (42) 15 (42) 6 (17)	3 (38) 4 (50) 1 (13)	6 (43) 7 (50) 1 (7)
Number of prior regimens, median (range)	–	3 (1–5)	3 (2–4)	3 (1–5)	3.5 (3–5)	3 (2–5)
Prior therapy, n (%) Anti-CD20 Platinum Anthracycline Bendamustine Lenalidomide Proteasome inhibitor ASCT BTKi therapy Ibrutinib Acalabrutinib Both	10 (100) 1 (10) 10 (100) 4 (40) 1 (10) 4 (40) 4 (40) 10 (100) 10 (100) 2 (20) 2 (20)	68 (100) 16 (24) 49 (72) 37 (54) 19 (28) 25 (37) 29 (43) 68 (100) 58 (85) 16 (24) 6 (9)	17 (100) 2 (12) 14 (82) 5 (29) 3 (18) 8 (47) 8 (47) 17 (100) 17 (100) 5 (29) 5 (29)	36 (100) 11 (31) 26 (72) 20 (56) 11 (31) 12 (33) 15 (42) 36 (100) 30 (83) 7 (19) 1 (3)	8 (100) 1 (13) 5 (63) 6 (75) 4 (50) 2 (25) 3 (38) 8 (100) 5 (63) 3 (38) 0	1 (100) 6 (43) 11 (79) 7 (50) 1 (7) 3 (21) 6 (43) 14 (100) 13 (93) 2 (14) 1 (7)
Received bridging therapy, n (%)	–	25 (37)	3 (18)	14 (39)	5 (63)	7 (50)
Response to last regimen, n (%) Complete response Partial response Stable disease Progressive disease Unknown	– – – – –	6 (9) 6 (9) 6 (9) 20 (29) 1 (1)	2 (12) 2 (12) 0 5 (29) 0	3 (8) 3 (8) 4 (11) 10 (28) 1 (3)	0 1 (13) 0 4 (50) 0	1 (7) 0 3 (21) 3(21) 1 (7)
Median tumor burden (SPD) by central read (mm^2^),^g^ median (range)	1906.6 (177–21446)	2087.7 (260–16878)	709.7 (260–5722)	2848.6 (340–16878)	737.4 (293–2815)	2166.5 (669–10624)
Positive bone marrow assessment at baseline, n (%)	4 (40)	37 (54)	10 (59)	16 (44)	8 (100)	8 (57)

^a^ Data cutoff October 05, 2023. Not all data are available for this patient group. ^b^ Data cutoff December 31, 2020. ^c^ Data cutoff April 01, 2024

^d^ Data cutoff July 24, 2019. ^e^ “Other” include “Kappa light chain restricted mantle cell lymphoma” and “Mantle cell lymphoma, Aggressive Variant”

^f^Ki-67 (%) IHC by central laboratory represents percent of tumor cells stained positively for Ki-67. ^g^As measured by the sum of the products of diameters of all target lesions at baseline. For patients who had bridging therapy, the measurement on or after bridging therapy end date is used as baseline

ASCT, autologous stem cell transplantation; Axi-cel, axicabtagene ciloleucel; brexu-cel, brexucabtagene autoleucel; BTKi, Bruton tyrosine kinase inhibitor; ECOG, Eastern Cooperative Oncology Group; FISH, fluorescence in situ hybridization; IHC, immunohistochemistry; LDH, lactate dehydrogenase; MCL, mantle cell lymphoma; MIPI, Mantle Cell Lymphoma International Prognostic Index; SPD, sum of the products of diameters; ULN, upper limit of normal

### Efficacy update in Brexu-cel–treated patients

The primary analysis of Cohort 1 efficacy was previously reported. [2] The ORR in Cohort 2 was 93% (95% CI, 66.1–99.8, as assessed by IRRC; *N* = 14; data cutoff date, July 24, 2019) with a CR in 64% (95% CI, 35.1–87.2) of patients and a PR in 29% (95% CI, 8.4–58.1) of patients (Table 2). No patients had SD or PD, and 1 patient was not assessed at the time of the analysis. There was a high concordance with investigator-assessed BOR rates (Table 2).

**Table 2:** BOR in Cohort 2

*n*, (%)	IRRC-assessed (*N* = 14)	Investigator-assessed (*N* = 14)
Objective response	13 (93)	12 (86)
Complete response	9 (64)	8 (57)
Partial response	4 (29)	4 (29)
Stable disease	0	1 (7)
Disease progression	0	0
Not assessed	1 (7)	1 (7)

Data cutoff date: July 24, 2019

BOR, best overall response; IRRC, independent radiology review committee

Five patients in Cohort 1 (4 CR and 1 not estimable [NE]) and 1 patient in Cohort 2 (CR) received a second infusion of brexu-cel following PD (Table S2). Best response to retreatment included 1 CR, 3 PRs, 1 NE, and 1 PD.

**Fig. 1:**
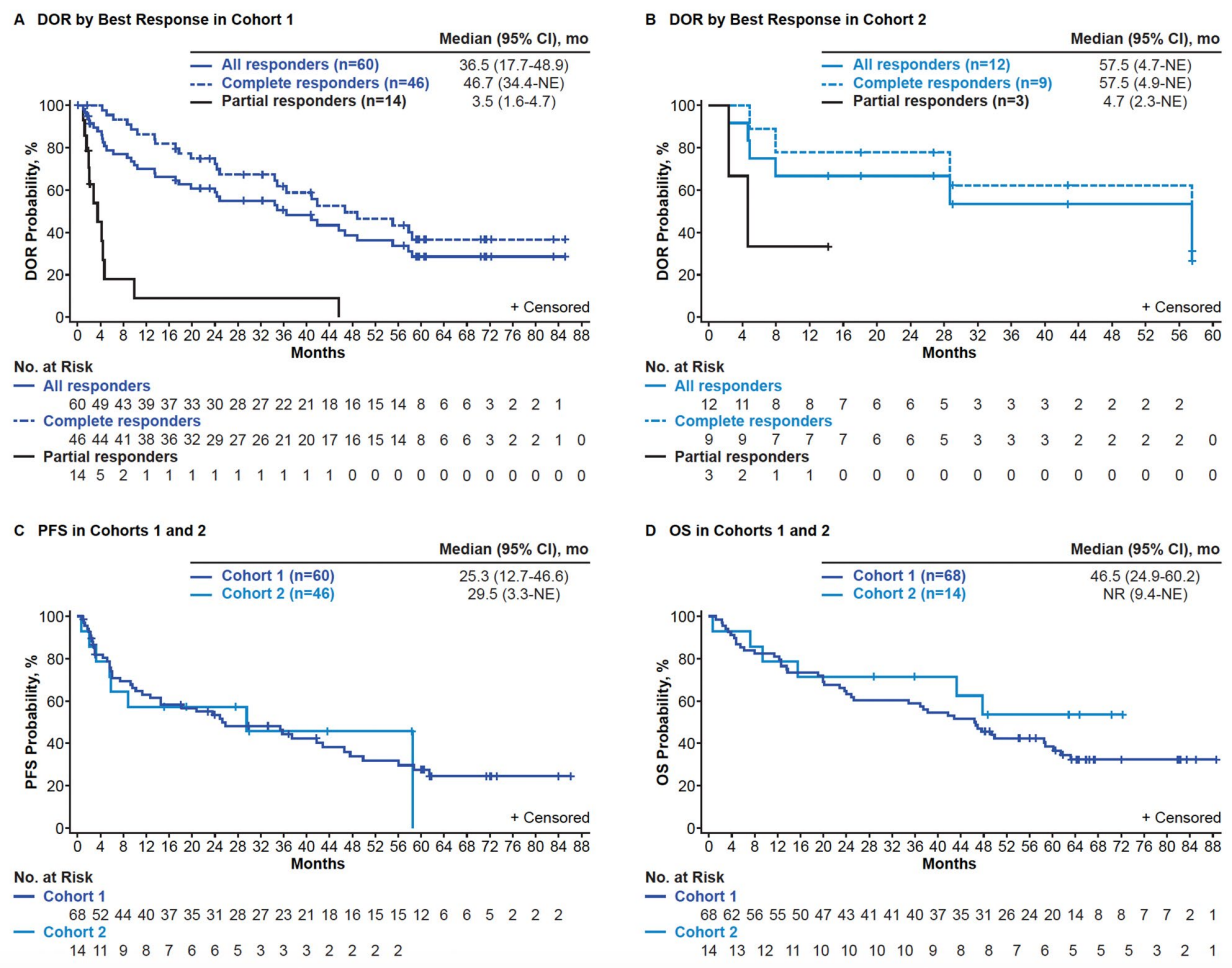
5-Year Analysis of DOR, PFS, and OS in ZUMA-2. Depicted are DOR per investigator assessment by best response (CR and PR) in Cohort 1 (**A**) and 2 (**B**), PFS per investigator assessment in Cohorts 1 and 2 (**C**), and OS in Cohort 1 and 2 (**D**). Data cutoff April 01, 2024. Brexu‑cel, brexucabtagene autoleucel; CR, complete response; DOR, duration of response; IRRC, independent radiology review committee; NE, not estimable; OS, overall survival; PFS, progression-free survival; PR, partial response

In the 5-year analysis, the median investigator-assessed DOR was 36.5 months in Cohort 1 (95% CI, 17.7–48.9; *n* = 60; Fig. 1A). Of the 60 responders at time of the DOR analysis, 24 patients had relapsed (40%), 17 patients were censored for ongoing response without additional therapy (28%; all CR), 12 patients had died (20%), 3 patients were censored for starting subsequent anticancer therapy (5%), 2 patients were censored for proceeding to subsequent SCT (3%), and 2 patients were censored due to withdrawal of consent or lost to follow-up (3%). The median investigator-assessed DOR in patients with CR was 46.7 months (*n* = 46; 95% CI, 34.4- NE) and was 3.5 months (*n* = 14; 95% CI, 1.6–4.7) in patients with PR (Fig. 1A). Among relapsed responders (*n* = 36) the median DOR was 1 3.6 months (95% CI, 5.1–24.3).

In Cohort 2, the median investigator-assessed DOR was 57.5 months (95% CI, 4.7-NE; *n* = 12; Fig. 1B). Of the 12 responders, at time of the DOR analysis, 6 patients had relapsed (50%), 3 patients were censored for ongoing response (25%; all CR), 2 patients were censored due to withdrawal of consent or lost to follow-up (17%), and 1 patient was censored for starting subsequent anticancer therapy (8%). Median investigator-assessed DOR in patients with CR was 57.5 months (*n* = 9; 95% CI, 4.9-NE) and was 4.7 months (*n* = 3; 95% CI, 2.3-NE) in patients with PR.

Median investigator-assessed PFS was 25.3 months (95% CI, 12.7–46.6; *N* = 68) in Cohort 1 and 29.5 months (95% CI, 3.3-NE; *N* = 14) in Cohort 2 (Fig. 1C). The 54-month PFS rate was 32% (95% CI, 20.0–44.2.0.2) for Cohort 1 and 46% (95% CI, 17.3–70.5) for Cohort 2.

**Fig. 2:**
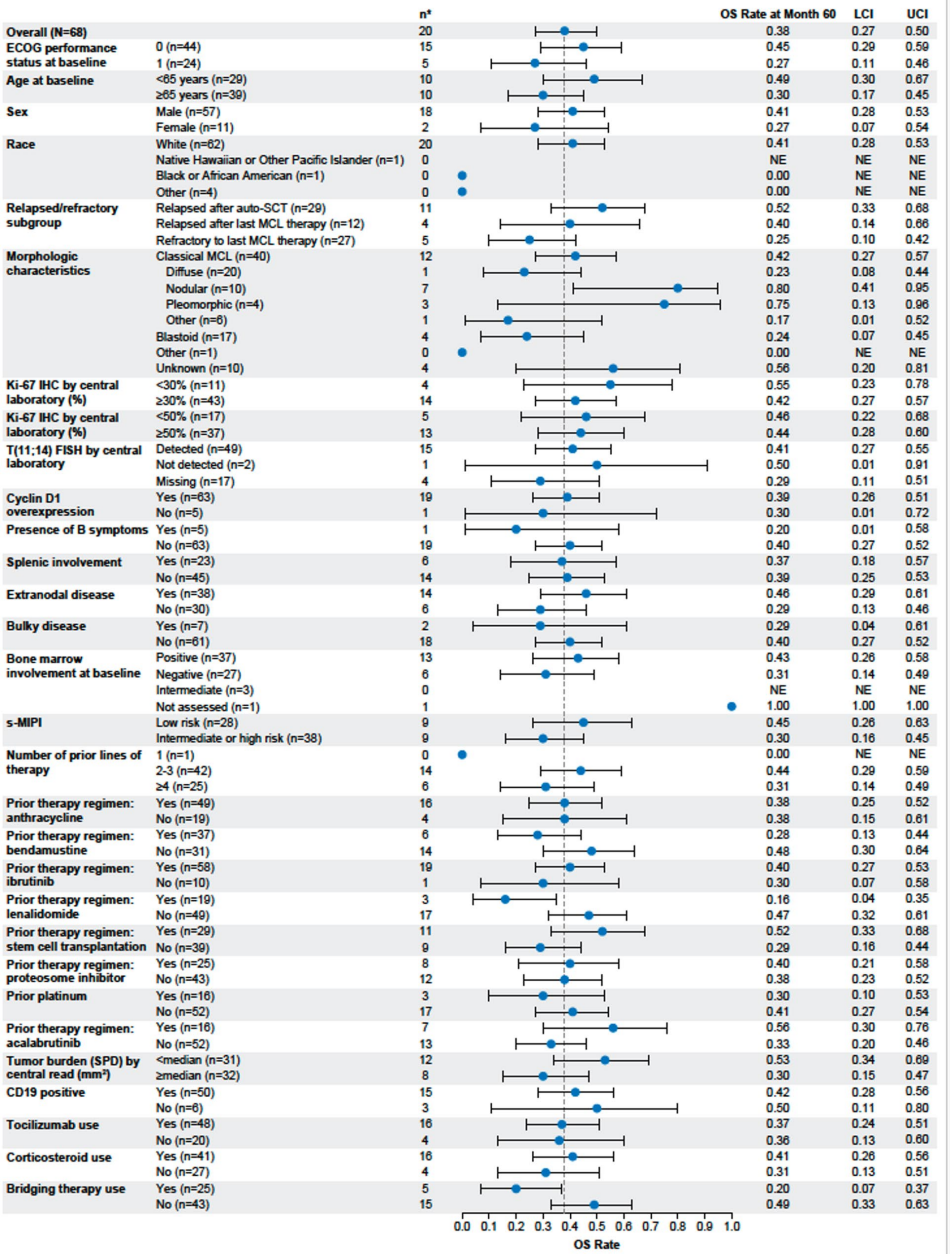
5-year OS rate by key subgroups in Cohort 1. The forest plot shows the analysis of the 5-year OS rate according to key demographic and baseline characteristics. The 95% confidence intervals were calculated using the Clopper–Pearson method. Data cutoff April 01, 2024. Auto-SCT, autologous stem cell transplantation; ECOG, Eastern Cooperative Oncology Group; FISH, fluorescence in situ hybridization; IHC, immunohistochemistry; LCI, lower confidence interval; MCL, mantle cell lymphoma; NE, not estimable; OS, overall survival; s-MIPI, simplified Mantle Cell Lymphoma International Prognostic Index; SPD, sum of the products of diameters; UCI, upper confidence interval

In Cohort 1, the median OS was 46.5 months (95% CI, 24.9–60.2; Fig. 1D) and the 60-month OS rate was 38% (95% CI, 26.7–50.1). Median OS was 60.2 months (*n* = 46; 95% CI, 42.8-NE) in patients with CR, 16.3 months (95% CI, 3.8–46.6) in patients with PR (*n* = 16), and 8.5 months (95% CI, 2.3-NE) in patients with no response (*n* = 6; IRRC-assessed; Figure S5A). Median OS was 35.9 months (*n* = 36; 95% CI, 20.1–46.6) for relapsed responders. In Cohort 1, OS rates at 60 months in key subgroups defined by ECOG performance status, morphologic subtype, Ki-67 expression, s-MIPI risk category, prior stem cell transplantation status, and tumor burden were generally within 10%−15% of that observed in the overall population (Fig. 2). Notable numerical differences were observed by prior lenalidomide status (yes, 16%; no, 47%), prior bendamustine status (yes, 28%; no, 48%), prior SCT status (yes, 52%, no, 29%), and prior bridging therapy status (yes, 20%; no, 49%, respectively; Fig. 2). This subgroup analysis was descriptive in nature, and no formal statistical testing was performed. In Cohort 2, median OS was not reached (NR; 95% CI, 9.4-NE; *N* = 14) and the 60-month OS rate was 54% (95% CI, 23.8–76.2; Fig. 1D). Median OS was NR (95% CI, 15.5-NE) in patients with CR (*n* = 9), was 28.6 months (95% CI, 7.2-NE) in patients with PR (*n* = 4), and was 0.6 months (NE-NE) in the 1 patient with no response (IRRC-assessed; Figure S5B).

### Safety update in ZUMA-2 patients

All 10 patients treated with axi-cel experienced at least one Grade ≥ 3 AE (Table 3). The most common Grade ≥ 3 AEs were cytopenias: thrombocytopenia/platelet count decreased (90%), neutropenia (80%), and anemia (70%). All patients experienced cytokine release syndrome (CRS) of any Grade, with 20% experiencing Grade ≥ 3, and 80% of patients experienced neurologic events, with 50% experiencing Grade ≥ 3. Axi-cel–treated patients had numerically higher rates of Grade ≥ 3 CRS, neurologic events, thrombocytopenia, and anemia than brexu-cel–treated patients (Table 3). Two patients developed subsequent primary malignancies (myelodysplastic syndromes [MDS]/acute myeloid leukemia [AML] [*n* = 1] and prostate cancer [*n* = 1]; Table S3). No cases of subsequent T-cell malignancies were reported in axi-cel–treated patients in Cohort 1 prior to this analysis.

**Table 3:** Summary of AEs of special interest in Cohorts 1 and 2

AE of special interest	Cohort 1 axi-cel^a^ (*N* = 10)	Cohort 1 brexu-cel^a^ (*N* = 68)	Cohort 2 brexu-cel^a^ (*N* = 14)
Any TEAE, n (%) Worst Grade ≥ 3, n (%)	10 (100) 10 (100)	68 (100) 67 (99)	14 (100) 13 (93)
Any CAR T cell-related TEAE, n (%) Worst Grade ≥ 3, n (%)	10 (100) 9 (90)	66 (97) 54 (79)	14 (100) 10 (71)
Any SAE, n (%) CAR T cell-related SAE, n (%)	9 (90) 7 (70)	49 (72) 37 (54)	9 (64) 7 (50)
Any CRS^b^, n (%) Worst Grade ≥ 3, n (%)	10 (100) 2 (20)	62 (91) 10 (15)	13 (93) 2 (14)
Any neurological events^c^, n (%) Worst Grade ≥ 3, n (%)	8 (80) 5 (50)	43 (63) 21 (31)	13 (93) 6 (43)
Any cytopenia^d^, n (%) Worst Grade ≥ 3, n (%)	10 (100) 10 (100)	65 (96) 64 (94)	11 (79) 11 (79)
Any thrombocytopenia^d^, n (%) Worst Grade ≥ 3, n (%)	10 (100) 9 (90)	50 (74) 36 (53)	7 (50) 6 (43)
Any neutropenia^d^,n (%) Worst Grade ≥ 3, n (%)	8 (80) 8 (80)	59 (87) 58 (85)	11 (79) 11 (79)
Any anemia^d^, n (%) Worst Grade ≥ 3, n (%)	10 (100) 7 (70)	47 (69) 36 (53)	7 (50) 6 (43)
Any infection^d^, n (%) Worst Grade ≥ 3, n (%)	4 (40) 2 (20)	37 (54) 26 (38)	7 (50) 3 (21)
Hypogammaglobulinemia^d^, n (%) Worst Grade ≥ 3, n (%)	1 (10) 0	14 (21) 1 (1)	0 0

^a^Data cutoff October 05, 2023.^b^CRS events are graded per the revised grading system proposed by Lee, et al. 2014. ^c^Neurologic events are identified based on Topp, et al. 2015. [9]

^d^All other events are graded per CTCAE version 4.03

AE, adverse event; Axi-cel, axicabtagene ciloleucel; brexu-cel, brexucabtagene autoleucel; CAR, chimeric antigen receptor; CRS, cytokine release syndrome; CTCAE, Common Terminology Criteria for Adverse Events; SAE, serious adverse event; TEAE, treatment-emergent adverse event

Treatment-emergent AEs that occurred in Cohort 1 in patients treated with brexu-cel were previously reported. [2, 3], Grade ≥ 3 AEs by preferred term were mostly hematologic in nature. The most common Grade ≥ 3 AEs for Cohorts 1 and 2 were anemia (51% and 43%), neutrophil count decreased (53% and 43%), hypotension (22% and 57%), platelet count decreased (38% and 36%), and white blood cell count decreased (41% and 50%; Table 4). Rates of Grade ≥ 3 CRS and neurologic events were 15% and 31% in Cohort 1, and 14% and 43% in Cohort 2, respectively (Table 3). There were no Grade 5 CRS or neurologic events observed up to time of analysis. All CRS events resolved within a median of 10 days (range, 1–50) in Cohort 1 and 10 days (range, 3–36) in Cohort 2. In Cohort 1, 93% (40/43) of neurologic events resolved with a median of 15 days to resolution (range, 1–708). In Cohort 2, 85% (11/13) of neurologic events resolved with a median of 17 days (range, 4–200) to resolution. Seven patients developed subsequent primary malignancies in Cohort 1 (MDS/AML [*n* = 4], non-melanoma skin cancer [*n* = 1], other malignancies [*n* = 2]), and 2 patient developed subsequent primary malignancies in Cohort 2 (MDS/AML [*n* = 1] and non-melanoma skin cancer [*n* = 1]; Table S3). No subsequent brexu-cel–related T-cell malignancies were reported at any time in ZUMA-2. No patient was positive for replication-competent retrovirus at any time on study.

**Table 4:** Most common TEAEs by preferred term in brexu-cel–treated patients

TEAEs occurring in ≥ 40% in either cohort, *n* (%)^a^	Cohort 1 (*N* = 68)	Cohort 2 (*N* = 14)
Any TEAE Grade ≥ 3	68 (100) 67 (99)	14 (100) 13 (93)
Any brexu‑cel–related TEAE Grade ≥ 3	66 (97) 54 (79)	14 (100) 10 (71)
TEAEs in ≥ 40% of patients in either cohort		
Any pyrexia Grade ≥ 3	64 (94) 9 (13)	13 (93) 3 (21)
Any anemia Grade ≥ 3	46 (68) 35 (51)	7 (50) 6 (43)
Any neutrophil count decreased Grade ≥ 3	37 (54) 36 (53)	6 (43) 6 (43)
Any hypotension Grade ≥ 3	36 (53) 15 (22)	11 (79) 8 (57)
Any platelet count decreased Grade ≥ 3	35 (51) 26 (38)	5 (36) 5 (36)
Any chills Grade ≥ 3	28 (41) 0	6 (43) 0
Any white blood cell count decreased Grade ≥ 3	28 (41) 28 (41)	7 (50) 7 (50)
Any fatigue Grade ≥ 3	26 (38) 1 (1)	7 (50) 0
Any hypoxia Grade ≥ 3	26 (38) 14 (21)	7 (50) 2 (14)
Any tremor Grade ≥ 3	24 (35) 0	7 (50) 2 (14)
Any nausea Grade ≥ 3	22 (32) 1 (1)	7 (50) 0
Any decrease in appetite Grade ≥ 3	15 (22) 0	7 (50) 0
Any confusional state Grade ≥ 3	14 (21) 8 (12)	6 (43) 1 (7)
Any dyspnea Grade ≥ 3	14 (21) 2 (3)	6 (43) 3 (21)

^a^Data cutoff October 05, 2023. TEAEs are defined as any AE with onset on or after initiation of brexu‑cel infusion. AEs that occurred on/after retreatment are not included. AEs are coded using MedDRA version 26.0 and graded per CTCAE version 4.03. Multiple incidences of the same AE in 1 patient are counted once at the highest grade for that patient

AE, adverse event; brexu‑cel, brexucabtagene autoleucel; CTCAE, Common Terminology Criteria for Adverse Events; MedDRA, Medical Dictionary for Regulatory Activities; TEAE, treatment‑emergent adverse event

**Fig. 3:**
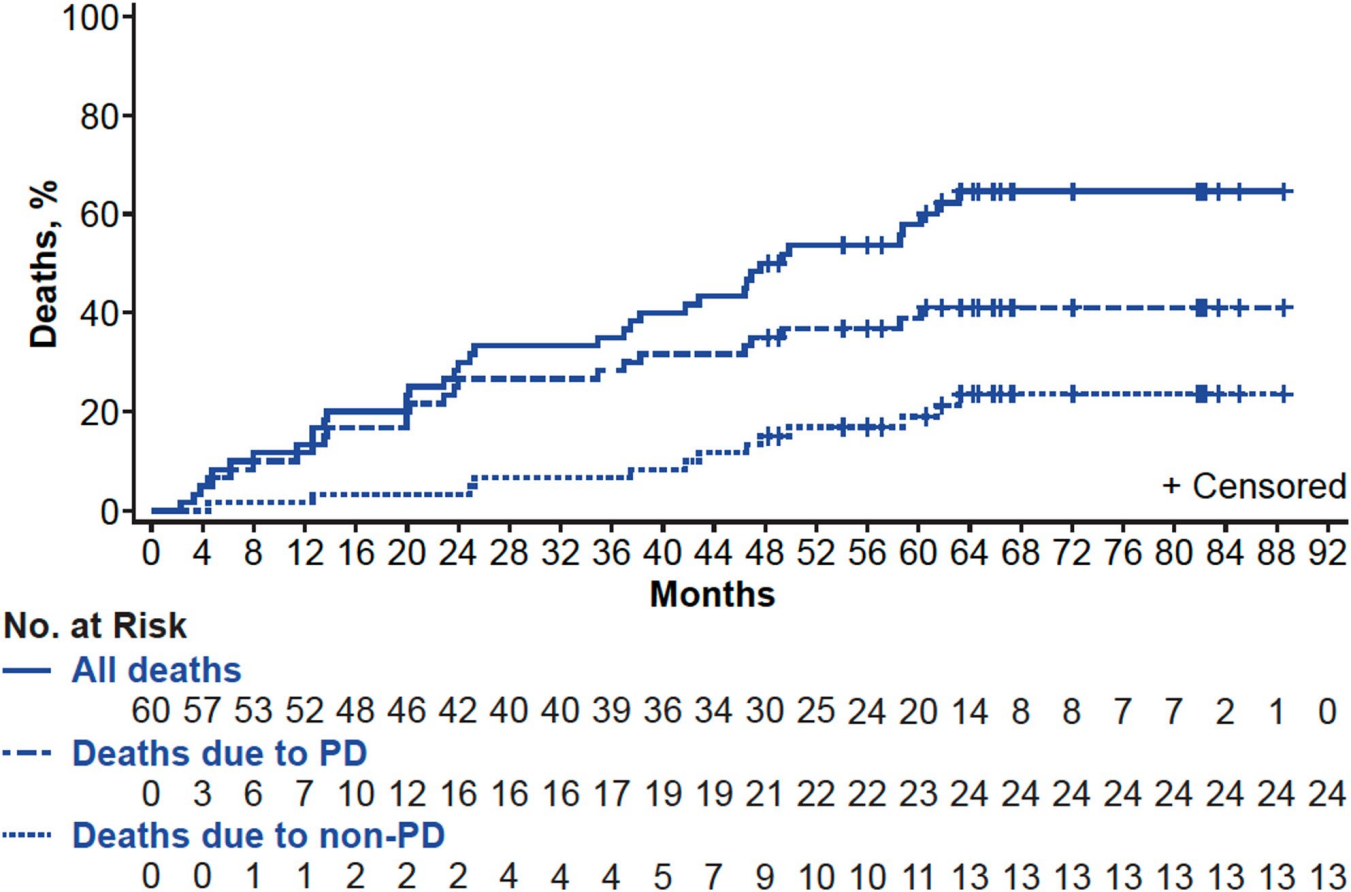
Cumulative incidence of relapse-related and non-relapse–related mortality in Cohort 1 responders. The graph depicts Kaplan-Meier estimates of cause specific mortality. Data cutoff April 01, 2024. PD, progressive disease

At data cutoff, 5 of the 10 axi-cel–treated patients had died with 4 deaths due to PD and 1 due to other causes (unknown cause; Table S4). Of the 68 brexu-cel–treated Cohort 1 patients, 44 had died (65%), with 28 deaths due to PD (41%) and 16 due to other causes (24%). In Cohort 2, 6 of the 14 patients had died at data cutoff with 4 deaths due to PD and 2 due to other causes. Reasons for non-relapse–related mortality are detailed in Table S4. Using cumulative incidence function analysis with death due to PD as a competing risk, the estimated cumulative incidence of non-relapse mortality in Cohort 1 was 1.7% (95% CI, 0.1%−7.9%) at 1 year, 3.3% (95% CI, 0.6%−10.3%) at 2 years, 6.7% (95% CI, 2.1%−14.9%) at 3 years, 15.0% (95% CI, 7.3%−25.3%) at 4 years, and 19.0% (95% CI, 10.0%−30.1%) at 5 years (Fig. 3).

During LTFU, 1 patient had 3 ongoing AEs: hypogammaglobulinemia and 2 viral infections that arose prior to LTFU. Two patients died on LTFU, both due to PD.

### Biomarker analysis

**Fig. 4:**
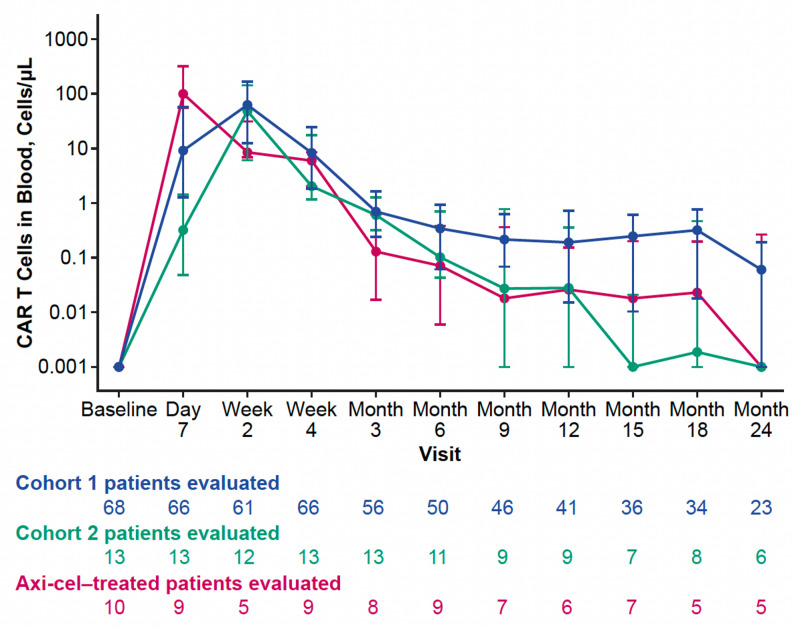
CAR T-cell expansion over time in all treated patients in ZUMA-2. The graph depicts CAR T-cell expansion and persistence over time in Cohort 1 (2 × 10^6^ anti-CD19 CAR T cells/kg) and Cohort 2 (0.5 × 10^6^ anti-CD19 CAR T cells/kg) patients treated with brexu-cel and Cohort 1 patients treated with axi-cel (2 × 10^6^ anti-CD19 CAR T cells/kg). Data cutoff April 01, 2024 Axi-cel; axicabtagene ciloleucel; CAR, chimeric antigen receptor; IQR, interquartile range

Median peak and area under the curve from Day 0–28 (AUC_0–28_) CAR T-cell levels were numerically higher in axi-cel–treated patients than in brexu-cel–treated patients, with a quicker median time to peak in the former (8 vs. 15 days, respectively; Table S5). Median peak and AUC_0–28_ CAR T-cell levels were numerically higher in patients who received the pivotal Cohort 1 dose of brexu-cel compared with those who received the Cohort 2 dose, whereas median time to peak was 15 days for both (Table S5). As previously reported for Cohort 1, median CAR T-cell levels continued to diminish over time after peak and fell below 1 cell/µL in the blood for all patient groups by 3 months post-infusion (Fig. 4). [3]

A post hoc exploratory analysis of correlations between product characteristics and PFS found that a higher percentage (> median) of naive/juvenile cells (CD3 + T cells expressing CCR7 and CD45RA) in the product prior to infusion trended toward an association with improved PFS in Cohort 1. However, this difference was not statistically significant (*P*=.096; Figure S6).

A post hoc exploratory analysis of peripheral blood B-cell levels in evaluable patients with (*n* = 25) and without (*n* = 30) Grade ≥ 3 infections anytime on study found that the median percentage of B cells in PBMCs were similar between both patient groups across time with diminished median B cell levels observed from Months 3–18 (Figure S7). The percentage of evaluable patients with B-cell aplasia (defined as having a B-cell level below the quantitation limit of the assay of 0.017%) was also similar between patient groups, ranging from 16% (4/25) and 17% (5/30) at baseline, to 55% (12/22) and 39% (11/28) at Month 3, and 53% (9/17) and 53% (8/15) at Month 18, respectively. Of note, lower B-cell levels and recovery at 24 months were observed in patients with Grade ≥ 3 infections, though not statistically significant.

## Discussion

Here we report 5-year outcomes in the pivotal ZUMA-2 study with the longest follow-up of an approved CAR T-cell therapy in adults with R/R MCL reported to date, to our knowledge. This study assessed the long-term outcomes of treatment with CAR T-cell therapy in patients with R/R MCL, including 68 patients in Cohort 1 who were treated with brexu-cel at the pivotal dose of 2.0 × 10^6^ anti-CD19 CAR T cells/kg, 10 patients in Cohort 1 who were treated with axi-cel at the same dose, and 14 patients in Cohort 2 who were treated with brexu-cel at a lower dose of 0.5 × 10^6^ anti-CD19 CAR T cells/kg. Given the small sample sizes and exploratory nature of the axi-cel subgroup and Cohort 2, only descriptive statistics were reported herein with no hypothesis or comparisons across cohorts intended.

Overall, patients in the pivotal ZUMA-2, Cohort 1 study experienced significant long-term benefits from brexu-cel therapy with a median DOR, per investigator, of 36.5 months and median OS of 46.5 months in a heavily pretreated R/R MCL patient population. As expected, patients with CR (*n* = 46) experienced the greatest benefit with a median DOR of 46.7 months and median OS of 60.2 months. After > 5 years of median follow-up, one-quarter of the treated patients (17/68) were still in ongoing response without additional therapies, all of whom had achieved a CR as their best response, suggesting brexu-cel is capable of inducing long-term remissions in a subset of patients with R/R MCL. OS benefits were demonstrated across key subgroups of interest including patients with high-risk features, although lower 60-month OS rates were observed for patients with (vs without) prior lenalidomide, bendamustine, or bridging therapies.

It should be noted that the long-term DOR analyses were based on investigator assessment rather than IRRC evaluation, as all patients had completed the prespecified IRRC assessment period at the time of the analysis. Although investigator assessment may introduce potential bias compared with independent review, the number of responders were similar between IRRC (*n* = 62) and investigator review (*n* = 60), which may contribute to minor differences in DOR estimates.

Similar to ZUMA-2 Cohort 3 findings, a post hoc exploratory analysis demonstrated a trend toward association between PFS with higher percentage (> median) of naive/juvenile cells in the product prior to infusion for Cohort 1; however, the results were not statistically significant for Cohort 1 while they were for Cohort 3. [10]

The safety profile for brexu-cel in the LTFU study of ZUMA-2 was expected, with no new safety signals detected after 5 years of follow-up. Rates of Grade ≥ 3 CRS and neurologic events observed across brexu-cel– and axi-cel–treated patients were similar to previous reports [4]. There were no Grade 5 CRS or neurologic events in ZUMA-2, and all CRS and most neurological events resolved within a median of 10 days and 15–17 days, respectively. The Grade ≥ 3 infection rate in Cohort 1 was 38%. A post hoc exploratory analysis found depleted B-cell levels in both patients with and without Grade ≥ 3 infections; thus, additional factors beyond B-cell aplasia may contribute to infection rates post CAR T-cell therapy. Additional biomarker analyses are ongoing to further understand the mechanisms behind serious infection rates in patients after brexu-cel treatment.

No subsequent T-cell malignancies were reported at any time in ZUMA-2. These findings are corroborated by a retrospective analysis of a large safety database of patients who received axi-cel or brexu-cel demonstrating that subsequent T-cell malignancies following treatment with these CAR T-cell therapies occur at very low rates (0.1%). [11] Of note, there were 6 cases of subsequent therapy-related myeloid neoplasms in ZUMA-2, exclusively occurring in patients with prior ASCT. Additionally, the observed 11% cumulative incidence of subsequent malignancies after 5 years of follow-up is concordant with prior real-world and clinical trial CAR T-cell studies, which have reported cumulative incidences of 6% to 14% after 22 to 36 months of follow-up in patients with R/R NHL, respectively.[12, 13]. These studies found that age, duration of follow-up, treatment setting, and number of prior therapies were associated with higher incidence of subsequent malignancies. [12, 13]. Therefore, the incidence of subsequent malignancies in this study likely reflects the cumulative impact of prior therapies in a heavily pretreated population (median prior number of therapies was 3), prior ASCT, patient-related risk factors such as advanced age, and extended follow-up time (> 5 years) rather than a attributable to CAR-T therapy itself. After 5 years of median follow-up, 35% of brexu-cel–treated patients in Cohort 1 were still alive; most deaths were due to PD and subsequent malignancies accounted for only 3 deaths (4%).

In Cohort 2, brexu-cel demonstrated a high ORR, and a safety profile consistent with prior ZUMA-2 studies. [2, 3]. Unsurprisingly, CAR T-cell expansion and persistence levels were lower in Cohort 2 patients, likely due to the lower dose. Given the small sample size and unmatched baseline characteristics, it is not feasible or advisable to compare Cohort 1 and 2 results. Therefore, these exploratory findings should not be interpreted as evidence supporting dose selection/reduction, cross-product comparisons, or modification of current manufacturing release standards.

Treatment with BTKi has demonstrated high response rates in clinical trials for patients with R/R MCL and has become a standard of care [14–16]. In the United States, acalabrutinib and zanubrutinib are BTKis approved for adults with R/R MCL with one or more prior lines of therapy, and ibrutinib is a BTKi approved in the EU for adults with R/R MCL whose disease has not responded to or has come back after treatment. [14, 15, 17] However, outcomes for patients who relapse after BTKi treatment are poor, with a median OS of 11–24 months after discontinuing BTKi [18, 19]. Therefore, a need for other salvage therapies exists. ZUMA-2 has demonstrated impressive outcomes in a heavily pretreated, post-BTKi patient population that would otherwise have had a poor prognosis. These long-term results are unmatched by any other therapy in this setting. In fact, lisocabtagene maraleucel, another autologous CD-19 CAR T-cell therapy recently approved in R/R MCL in the United States (after ≥ 2 lines of therapy including a BTKi) demonstrated similar ORR and CR rates to ZUMA-2 in patients with R/R MCL in the Phase 1 TRANSCEND NHL 001 study (86.5% and 74.3%, respectively); however, efficacy durability outcomes appeared less robust than in ZUMA-2 with a median DOR of 15.7 months (95% CI, 6.2–24.0; median follow-up of 22.8 months [95% CI, 16.7–23.0]) and median OS of 18.2 months (95% CI, 12.9–36.3; median follow-up of 24.0 months [95% CI, 23.7–24.2]). [20] Despite lower documented rates of Grade ≥ 3 CRS and neurologic events in TRANSCEND, the non-PD–related mortality rate after 16.1 months of follow-up was similar to the non-PD–related mortality rate in ZUMA-2 after > 5 years of median follow-up (19% and 24%, respectively). [20] Direct cross-trial comparisons should not be made, given differences in patient characteristics, study design, and other factors; nonetheless, no other therapy has demonstrated long-term survival similar to the remarkable benefits that brexu-cel has demonstrated in patients with R/R MCL.

## Conclusions

Together, these long-term outcomes in ZUMA-2 indicate that brexu-cel can induce durable responses with high OS rates, even in heavily pretreated patients with high-risk disease features. The long-term safety profile was predictable, with no new safety signals observed. The results of this study support the continued use of brexu-cel in patients with R/R MCL.

## Supplementary Information

Supplementary Material 1

## Data Availability

Kite is committed to sharing clinical trial data with external medical experts and scientific researchers in the interest of advancing public health, and access can be requested by contacting medinfo@kitepharma.com.

## Abbreviations

AE: Adverse event
AUC: Area under the curve
Axi-cel: Axicabtagene ciloleucel
BOR: Best objective response
Brexu-cel: Brexucabtagene autoleucel
BTKi: Bruton tyrosine kinase inhibitor
CAR: Chimeric antigen receptor
CR: Complete response
CRS: Cytokine release syndrome
DOR: Duration of response
IRRC: Independent radiology review committee
LTFU: Long-term follow-up
MCL: Mantle cell lymphoma
NE: Not estimable
NR: Not reached
ORR: Objective response rate
OS: Overall survival
PBMC: Peripheral blood stem cell
PD: Progressive disease
PFS: Progression-free survival
PR: Partial response
R/R: Relapsed/refractory
SAE: Serious adverse event
SCT: Stem cell transplantation
SD: Stable disease
