# Supplementary material for: Five-year follow-up of patients with relapsed/refractory mantle cell lymphoma treated with anti-CD19 CAR T-cell therapy in ZUMA-2, Cohorts 1 and 2
Source: J Hematol Oncol. 2026 Apr 27;19:39. doi: 10.1186/s13045-026-01797-4 (PMC13242663; doi:10.1186/s13045-026-01797-4)
Supplement: Supplementary file 1 — Supplementary Material 1 [file 13045_2026_1797_MOESM1_ESM.pdf]

Table of Contents

***Supplemental Appendix*......2**

Plain Language Summary......2

Supplemental Methods......4

Table S1. Late-onset AEs monitored in LTFU study......4

Supplemental Results......5

Table S2 Summary of patients with retreatment......5

Table S3 Summary of patients with subsequent malignancies......6

Table S4 Summary of causes of non-PD mortality in ZUMA-2......7

Table S5 Peak and $AUC_{0\text{-}28}$ CAR T-cell expansion in Cohort 1 and 2 patients......8

Fig. S1 ZUMA-2 Cohort 1 and 2 enrollment periods......9

Fig. S2 Disposition of Cohort 1 patients treated with axi-cel......10

Fig. S3 Disposition of Cohort 1 patients treated with pivotal brexu-cel dose......11

Fig. S4 Disposition of Cohort 2 patients treated with brexu-cel......12

Fig. S5 5-Year Analysis of OS in ZUMA-2......13

Fig. S6 PFS by median percentage of naive/juvenile cells in the product in Cohort 1......14

Fig. S7 Summary of B-cell levels by Grade ≥3 infections over time......15

Supplemental Appendix

Plain Language Summary

What is relapsed or refractory mantle cell lymphoma?

- Mantle cell lymphoma (MCL) is a type of blood cancer where white blood cells called B cells grow abnormally
- Relapsed or refractory MCL is a type of MCL that did not respond to previous treatment or came back after the previous treatment

Why was this study done?

- Brexucabtagene autoleucel (brexu-cel) is a one-time anticancer treatment called chimeric antigen receptor T-cell therapy and is made from a person's own immune cells
- ZUMA-2 is a clinical trial that looked at how safely and well brexu-cel worked in people with R/R MCL
- In this study, researchers looked at how safe and effective brexu-cel was in ZUMA-2 after 5 years of follow-up

How was the study conducted?

- Adults with R/R MCL received one treatment of brexu-cel and were then followed by researchers for over 5 years. Overall survival was calculated from the time when a person received treatment to when they died

What were the results of this study?

- After 5 years in ZUMA-2, 39% of people treated with brexu-cel were still alive and most people who had a complete response to brexu-cel lived longer than 5 years
- After 5 years, there were no new or unexpected side effects and more people died because their cancer got worse than for other reasons

What do these results mean?

- Brexu-cel continued to work well 5 years after treatment and can be an option for people whose MCL does not respond to, or comes back after, previous treatment

Supplemental Methods

Table S1. Late-onset AEs monitored in LTFU study

AE	Assessment
Neurologic disorders	Type, date of onset, severity, treatment, and date of resolution
Autoimmune disorders	Type, date of onset, severity, treatment, and date of resolution
Hematologic disorders	Type, date of onset, severity, treatment, and date of resolution
Serious infections[a]	Type, organism, and timing of infection
Subsequent malignancies	Time to development of the subsequent malignancy, type, location, and staging

[a]Viral, bacterial, or fungal.
AE, adverse event; LTFU, long-term follow-up.

Supplemental Results

Table S2 Summary of patients with retreatment

Cohort[a]	Best response to first infusion	Day of documented PD	Approximate time between infusions	Best response to retreatment
1	CR	Day 171	~7 months	PR
1	CR	Day 193	~12 months	CR
1	CR	Day 344	~13 months	PR
1	CR	Day 441	~15 months	PR
1	NE	Day 630	~22 months	NE
2	CR	Day 176	~9 months	PD

[a]All 6 patients received $2x10^6$ CAR T cells/kg on retreatment. CR, complete response; NE, not estimable; PD, progressive disease; PR, partial response.

Table S3 Summary of patients with subsequent malignancies

**Subsequent Malignancies**	***n***	**Study day**	**Related to study treatment by medical review/investigator**
**Patients in Cohort 1 treated with axi-cel with any subsequent malignancy**			
**Therapy-related MDS or therapy-related AML**	1	1740	No/No
**Prostate cancer**	1	1814	No/No
**Patients in Cohort 1 treated with brexu-cel with any subsequent malignancy**			
**Therapy-related MDS or therapy-related AML**	4	252<br>532<br>659<br>1211	No/No<br>No/Yes (CC)<br>No/Yes (CC)<br>No/No
**Basal cell carcinoma**	1	448	No/No
**Multiple myeloma**	1	926	No/No
**Lung carcinoma**	1	1191	No/No
**Patients in Cohort 2 treated with brexu-cel with any subsequent malignancy**			
**Squamous cell carcinoma**	1	366	No/No
**Therapy-related MDS or therapy-related AML**	1	940	No/Yes (brexu-cel)

Axi-cel, axicabtagene ciloleucel; CC, conditioning chemotherapy-related.

Table S4 Summary of causes of non-PD mortality in ZUMA-2

Causes of non-PD mortality	Study day	Related to study treatment by investigator
**Patients in Cohort 1 treated with axi-cel**		
**Unknown**	1848	No
**Patients in Cohort 1 treated with brexu-cel**		
**Adverse event**		
**Organizing pneumonia**	37	CC
**Staphylococcal bacteremia**	134	LK, CC, and BRE
**Sepsis**	695	CC and BRE
**Salmonella bacteremia**	757	No
**Acute myeloid leukemia**	1303	No
**Subsequent malignancy**		
**Therapy-related MDS or therapy-related AML**	768	No
**Acute myeloid leukemia**	1140	No
**Lung carcinoma**	1786	No
**Other**		
**Myocardial infarction**	383	No
**Sepsis**	612	No
**Stroke**	1270	No
**Unknown**	1416	No
**Covid-19**	1448	No
**Unknown**	1517	No
**Covid-19**	1869	No
**Lung infection/pneumonia**	1920	No

**Patients in Cohort 2 treated with brexu-cel**		
**Adverse event**		
**Cardiac arrest**	18	No
**Other**		
**AlloSCT–related toxicity**	286	No

AlloSCT, allogeneic stem cell transplantation; AML, acute myeloid leukemia; Axi-cel, axicabtagene ciloleucel; BRE, brexu-cel–related; CC, conditioning chemotherapy-related; LK, leukapheresis-related; MDS, myelodysplastic syndrome; PD, progressive disease.

Table S5 Peak and $AUC_{0\text{-}28}$ CAR T-cell expansion in Cohort 1 and 2 patients

	**Cohort 1[a] axi-cel (*N*=10)**	**Cohort 1[b] brexu-cel (*N*=68)**	**Cohort 2[c] (*N*=14)**
**Median $AUC_{0\text{-}28}$ (cells/ µL x days), (IQR)**	1441.37 (847.38-2375.26)	1112.86 (230.75-3005.32)	688.40 (286.72-1477.66)
**Median peak CAR T-cells in the blood (cells/ µL), (IQR)**	99.69 (54.79-318.03)	83.12 (17.40-265.71)	56.07 (26.34-139.16)
**Median time to peak (days), (IQR)**	8 (8-8)	15 (8-15)	15 (15-29)

[a] Data cutoff on February 1, 2019.
[b] Data cutoff on October 5, 2023.
[c] Data cutoff on October 5, 2023.
Axi-cel, axicabtagene ciloleucel; brexu-cel, brexucabtagene autoleucel; $AUC_{0\text{-}28}$, area under the curve from Day 0-28; CAR, chimeric antigen receptor; IQR, interquartile range.

Fig. S1 ZUMA-2 Cohort 1 and 2 enrollment periods

Axi-cel, axicabtagene ciloleucel; brexu-cel, brexucabtagene autoleucel.

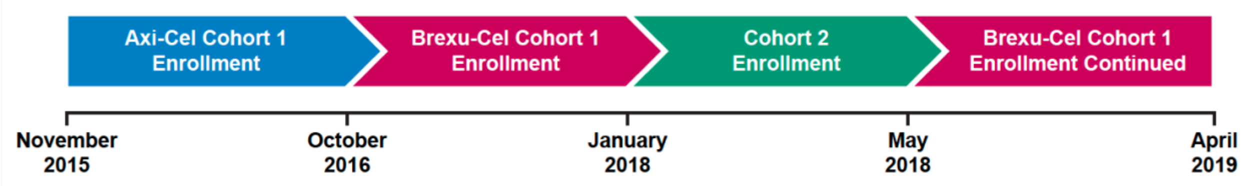

Fig. S2 Disposition of Cohort 1 patients treated with axi-cel

Data cutoff October 05, 2023.
Axi-cel, axicabtagene ciloleucel.

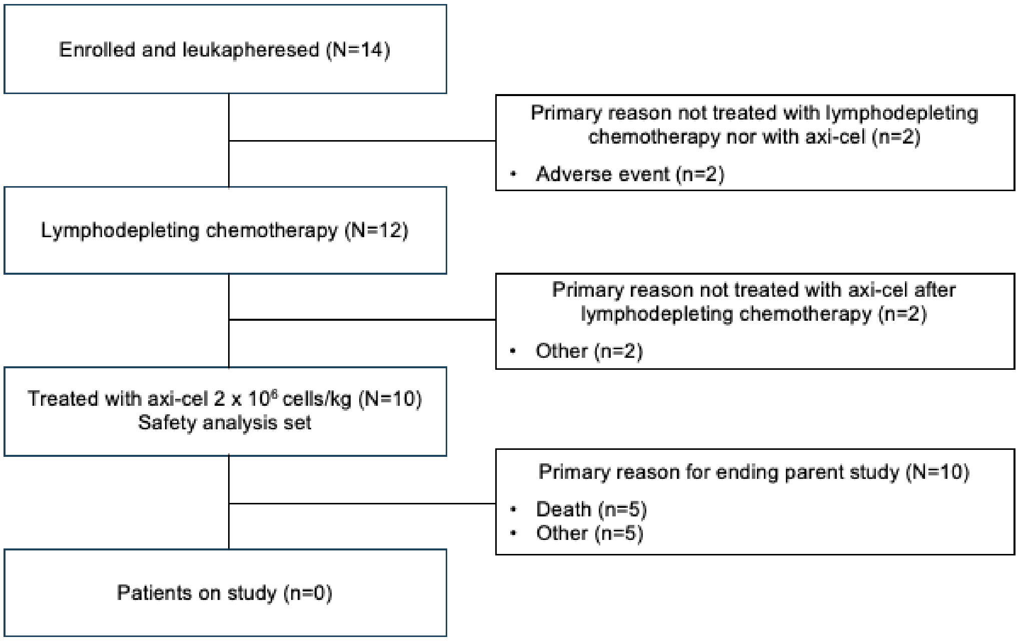

Fig. S3 Disposition of Cohort 1 patients treated with pivotal brexu-cel dose

Data cutoff April 01, 2024. First patient enrolled to LTFU on March 31, 2022.
[a] Brexu-cel was not successfully manufactured for 1 of the patients in Cohort 1 who died due to disease progression before having the opportunity to be leukapheresed a second time.
[b] Brexu-cel was not successfully manufactured for patient after 2 leukapheresis attempts.
[c] Brexu-cel was not successfully manufactured from patient's leukapheresis material; patient had AEs that precluded treatment before having the opportunity to be leukapheresed a second time.
[d] Patient did not meet criteria for infusion due to history of atrial fibrillation.
AE, adverse event; brexu-cel, brexucabtagene autoleucel; LTFU, long-term follow-up.

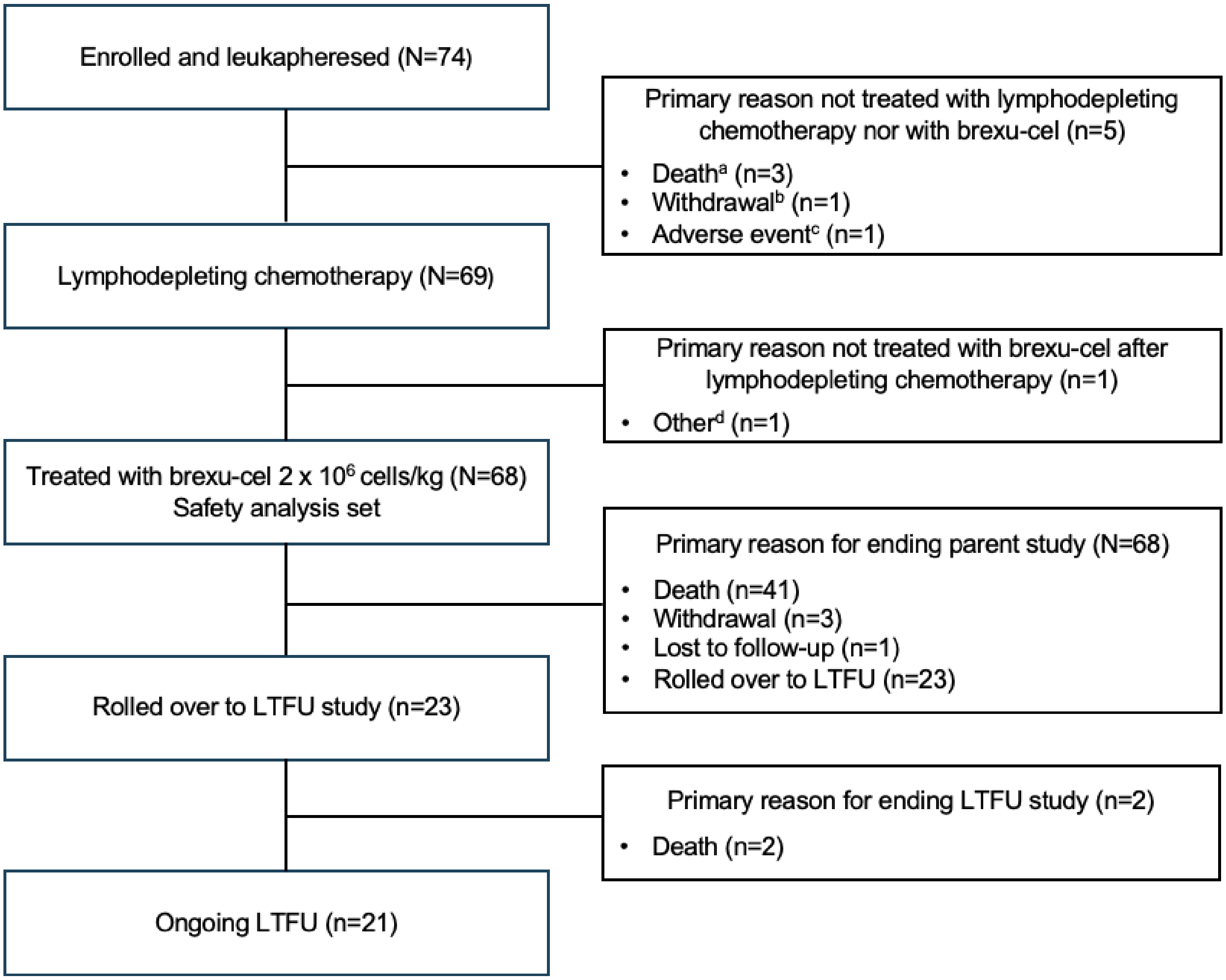

Fig. S4 Disposition of Cohort 2 patients treated with brexu-cel

Data cutoff April 01, 2024. First patient enrolled to LTFU on July 23, 2022.
[a] Brexu-cel was not successfully manufactured from patient's leukapheresis material; patient had AEs that precluded treatment before having the opportunity to be leukapheresed a second time.
[b] One patient was not enrolled into LTFU by data cutoff date but had initiated the process.
AE, adverse event; brexu-cel, brexucabtagene autoleucel; LTFU, long-term follow-up.

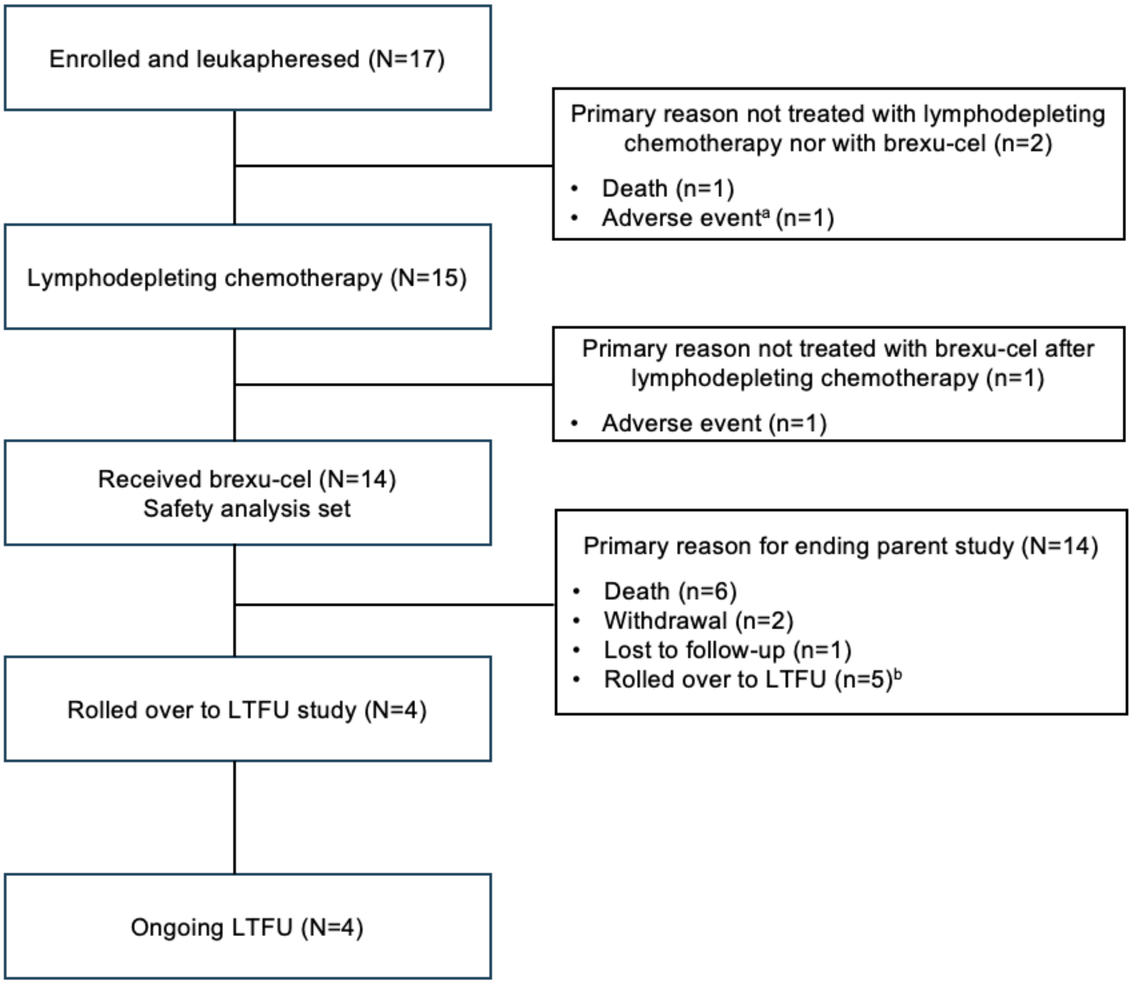

Fig. S5 5-Year Analysis of OS in ZUMA-2

The graphs show Kaplan-Meier estimates of OS by best response (CR and PR, per IRRC) in Cohort 1 (**A**) and 2 (**B**). Data cutoff April 01, 2024.
CR, complete response; IRRC, independent radiology review committee; NE, not estimable; OS, overall survival; PR, partial response.

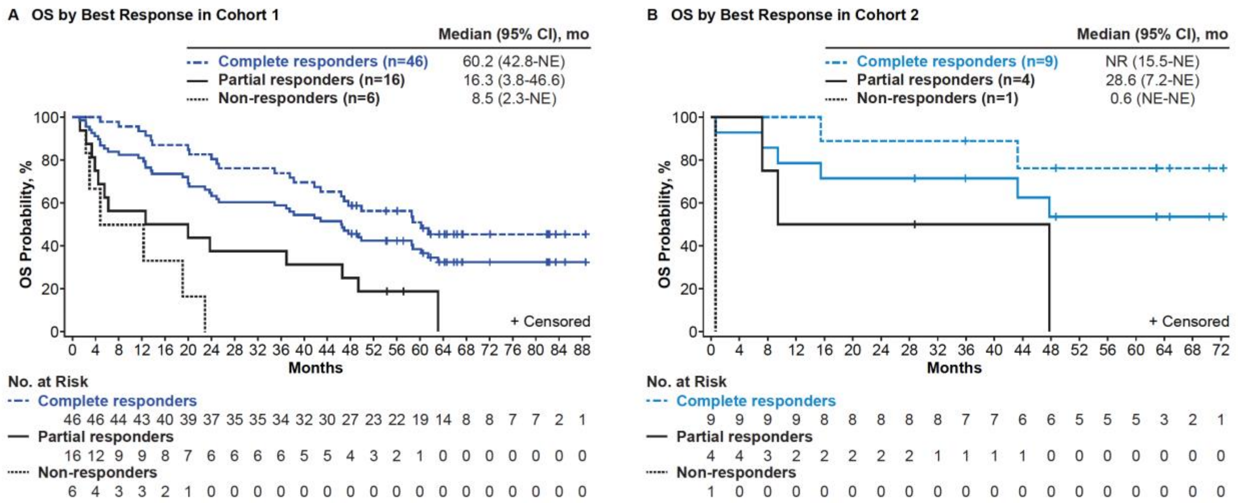

Fig. S6 PFS by median percentage of naive/juvenile cells in the product in Cohort 1

PFS was assessed per investigator. Median PNV was 24.45%. The *P* value was derived using the log-rank test with a threshold of 0.05, to be considered statistically significant.
PNV, percentage of naive cells in product.

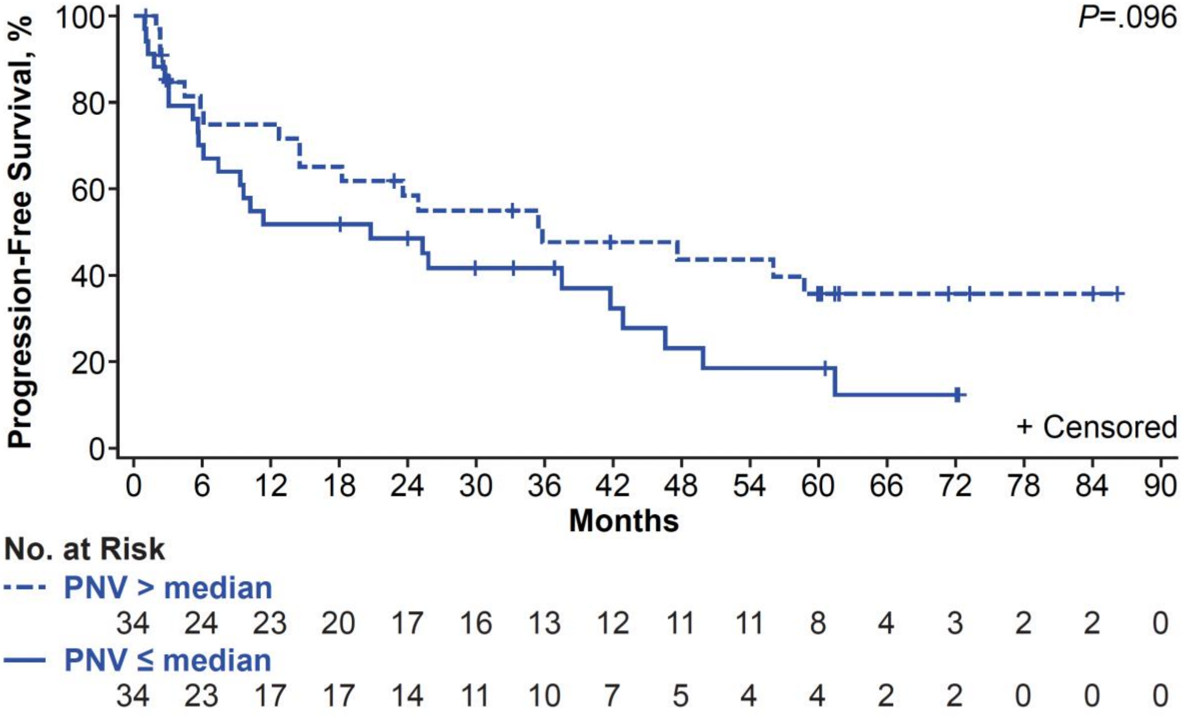

Fig. S7 Summary of B-cell levels by Grade ≥3 infections over time

PBMC, peripheral blood mononuclear cell.

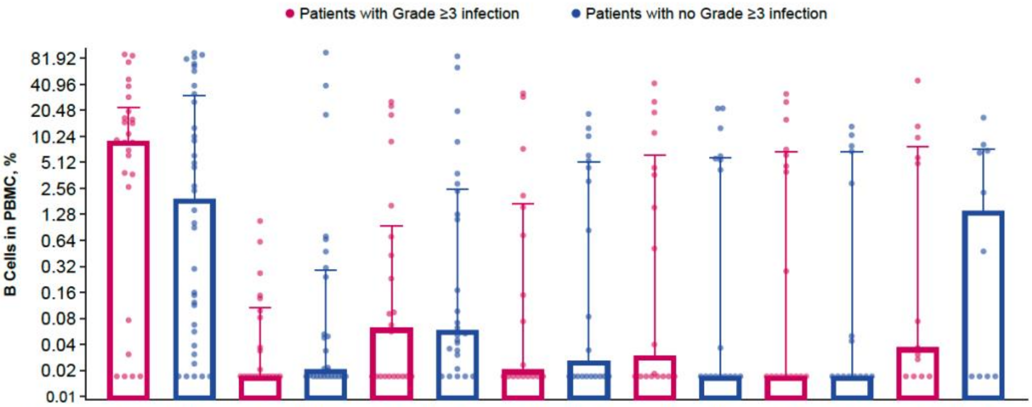

Months Post Infusion, Months	BASELINE	3 MONTHS	6 MONTHS	12 MONTHS	15 MONTHS	18 MONTHS	24 MONTHS
Patients with Grade ≥3 infection	25	22	22	18	18	17	13
Median % of B Cells in PBMC	8.750	0.017	0.06132	0.02003	0.02902	0.017	0.03637
Patients with no Grade ≥3 infection	30	28	26	20	17	15	10
Median % of B Cells in PBMC	0.9565	0.02044	0.05762	0.02558	0.017	0.017	1.385
